# RNA-Seq transcriptome analysis shows anti-tumor actions of melatonin in a breast cancer xenograft model

**DOI:** 10.1038/s41598-018-37413-w

**Published:** 2019-01-30

**Authors:** Bruna Victorasso Jardim-Perassi, Pâmela A. Alexandre, Nathalia M. Sonehara, Rubens de Paula-Junior, Osvaldo Reis Júnior, Heidge Fukumasu, Roger Chammas, Luiz Lehmann Coutinho, Debora Aparecida Pires de Campos Zuccari

**Affiliations:** 1 Faculdade de Medicina de São José do Rio Preto, Av. Brigadeiro Faria Lima, 5419, São José do Rio Preto, SP 15090-000 Brazil; 2 Universidade de São Paulo, Faculdade de Zootecnia e Engenharia de Alimentos, Av. Duque de Caxias Norte, 225 - Zona Rural, Pirassununga, SP 13635-900 Brazil; 3 Universidade Estadual de Campinas, Instituto de Biologia, Cidade Universitária Zeferino Vaz - Barão Geraldo, Campinas, SP 13083-970 Brazil; 4 Faculdade de Medicina da Universidade de São Paulo, Av. Dr. Arnaldo, 251 8th floor, São Paulo, SP 01246-000 Brazil; 5 Escola Superior de Agricultura Luiz de Queiroz, Av. Pádua Dias, 11 – Agronomia, Piracicaba, SP 13418-900 Brazil

## Abstract

Melatonin is a pleiotropic anti-cancer molecule that controls cancer growth by multiple mechanisms. RNA-Seq can potentially evaluate therapeutic response and its use in xenograft tumor models can differentiate the changes that occur specifically in tumor cells or in the tumor microenvironment (TME). Melatonin actions were evaluated in a xenograft model of triple-negative breast cancer. Balb/c nude mice bearing MDA-MB-231 tumors were treated with melatonin or vehicle. RNA-Seq was performed on the Illumina HiSeq. 2500 and data were mapped against human and mouse genomes separately to differentiate species-specific expression. Differentially expressed (DE) genes were identified and Weighted Gene Co-expression Network Analysis (WGCNA) was used to detect clusters of highly co-expressed genes. Melatonin treatment reduced tumor growth (p < 0.01). 57 DE genes were identified in murine cells, which represented the TME, and were mainly involved in immune response. The WGCNA detected co-expressed genes in tumor cells and TME, which were related to the immune system among other biological processes. The upregulation of two genes (Tnfaip8l2 and Il1f6) by melatonin was validated in the TME, these genes play important roles in the immune system. Taken together, the transcriptomic data suggests that melatonin anti-tumor actions occur through modulation of TME in this xenograft tumor model.

## Introduction

Breast cancer is the most common type of cancer in women^1^, while the triple-negative phenotype (negative for estrogen (ER), progesterone receptor (PR) and human epidermal growth factor receptor 2 (HER-2/neu)) has the poorest survival rate^2^. This subtype lacks specific targets^3^, remaining dependent on conventional chemotherapy, radiation, and surgery^4^. Thus, therapeutic agents that show effectiveness in triple negative breast cancer are of special interest.

Melatonin is a hormone synthesized mainly by the pineal gland and it is considered a “neuroendocrine translator” of the light-dark cycle, displaying several physiological functions^5^. Several anti-tumor actions were also described to melatonin, such as anti-proliferative^6^, proapoptotic^7^, antiangiogenic^8–11^, antimetastatic^12–14^, differentiation^15^ and antiestrogenic^16^, with multiple underlying mechanisms being proposed^17,18^. Some of the antimetastatic actions of melatonin involve the inhibition of cancer stem cells proliferation, as well as migration, matrix metalloproteinase 9 (MMP9) activity and expression of genes associated with epithelial-mesenchymal transition (EMT) in ovarian cancer cells^12^, as well as the upregulation of a suppressor of metastasis (kisspeptin) in MDA-MB-231 breast cancer cells^14^. In addition, the anti-angiogenic action of melatonin was evident in a co-culture system with human neuroblastoma cells and endothelial cells, where melatonin inhibited Vascular endothelial growth factor (VEGF) expression in the tumor cells and therefore, decreased the levels of the proangiogenic factor available for endothelial cells, reducing proliferation, migration and tube formation in the endothelial cells^8^. Melatonin can also attenuate angiogenesis by reducing TGFβ1, a transforming growth factor-beta1, hypoxia-inducible factor (HIF)-1α, VEGF and its receptor VEGFR2, which was demonstrated in an ovarian cancer DMBA-induced rat model^9^. HIF-1α and VEGF/VEGFRs reduction by melatonin were also observed in other tumor models^19–22^.

In breast cancer, melatonin efficacy is mainly described in ER-positive breast cancer, such as the MCF-7 cell line, in which physiological doses can exert anti-tumor effects^11^. The antiestrogenic effect of melatonin was demonstrated in a 7,12-dimethylbenza(a)anthracene (DMBA)-induced mammary tumor model, where melatonin treatment increased survival and inhibited the effects of estrone sulfate, a hormone that stimulates tumor growth in ovariectomized rats^16^. On the other hand, in triple-negative breast tumors, such as the MDA-MB-231 cell line, pharmacological doses of melatonin are needed to exert anti-tumor effects^11^. In previous studies, our group has shown several anti-tumor effects of melatonin in MDA-MB-231 models^10,11,13^. Melatonin can effect cells by binding to membrane receptors, MT1 and MT2, which are expressed in a variety of tissues, including breast and immune cells^23^. Melatonin can also act by receptor-independent mechanisms crossing the membrane and interacting with intracellular proteins and nuclear receptors RZR/ROR (retinoid Z receptor/orphan receptor for retinoid)^24^, producing antioxidants^25,26^ and an anti-inflammatory effect^27^. Both melatonin and its metabolites present important antioxidant properties, which are known to be an action independent of its receptors MT1 and MT2. It can protect melanocytes against UVB-induced reactive oxygen species (ROS) production, stimulating the expression of NRF2 (nuclear factor erythroid 2 [NF-E2]-related factor 2) and DNA-repair through the increase of p53 phosphorylated at Ser-15 expression^28^.

It is known that tumor biology is influenced by the microenvironment and the host immune response^4^. Immune cells in the tumor microenvironment (TME) can exert ambiguous functions during carcinogenesis, eliminating tumor cells, or, conversely promoting tumor growth^29–31^. A successful antitumor immune response requires many steps, involving not only the immune cells but also other components of the TME, such as the extracellular matrix (ECM), which serves as a physical barrier to prevent immune infiltration and promote immune escape^32^.

In this context, human tumor xenografts are commonly used to evaluate therapy response, as in these models the tumor growth is dependent on the interplay between the human tumor cells and murine stromal cells^33^. Athymic nude mice can be used to this purpose, as they have a T cell deficiency, allowing the growth of the xenografted tumor cells^34^. Although in this model the immunodeficiency is severe, it is not absolute, as the nude mutation allows an intact humoral adaptive immune system and an intact innate immune system^35^. In addition, despite the deficient immunologic environment, xenograft models preserve other important characteristics of the TME as the ECM, tumor stroma-associations, vascularization and a three-dimensional structure^35^, mimicking, at least to some extent, the biology in humans^34^.

Currently, RNA-Seq has spread well beyond the genomics community and has become a standard part of the research toolkit^36^. This technique can be used for different applications, such as the detection of differentially expressed (DE) genes and shows high accuracy. In addition to identifying DE genes, a weighted gene coexpression network analysis (WGCNA) has recently been proposed as a technique to explore the relationship between genes and identify intrinsic modules of coordinately expressed genes^37,38^. The use of RNA-Seq in xenograft samples can be a useful tool to potentially identify biomarkers and direct changes in response to therapy, distinguishing the human tumor cells from the TME, characterized by the murine cells^33^.

Thus, the aim of this study was to perform RNA-Seq transcriptomic analysis to evaluate the pathways involved in the melatonin anti-tumor actions in MDA-MB-231 breast cancer xenograft model. In a previous study, our group showed that melatonin treatment was able to reduce tumor growth in this xenograft model, presenting anti-angiogenic effects^11^. Thus, this study was developed to investigate the genome-wide transcriptional anti-tumor melatonin responses in a triple negative breast cancer model, through RNA-Seq analysis.

## Results

### Melatonin treatment controls the tumor growth

**Figure 1:**
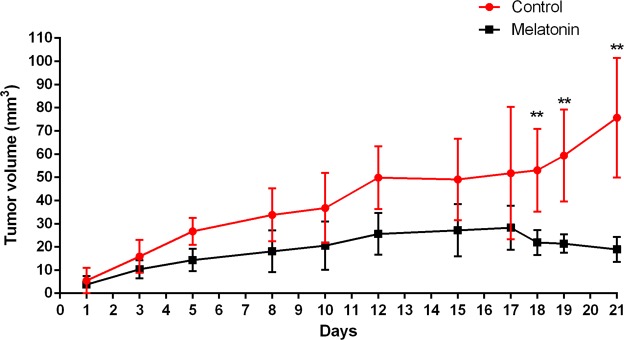
Melatonin treatment reduced tumor growth in MDA-MB-231 xenograft model. Tumor volume was monitored by caliper for 21 days. Tumor growth was calculated and compared between groups for each time-point. **p < 0.01; Statistically significant difference between melatonin-treated and control groups using Student’s t-test.

Athymic nude mice were inoculated with MDA-MB-231 human breast tumor cells and treated with melatonin (40 mg/kg) or vehicle (control). Treatments were administrated by IP injection 5 days a week for 21 days. Results showed that melatonin treatment reduced the tumor growth when compared with mice treated with vehicle. The mean tumor volume was significantly smaller in the melatonin-treated group from day 18 until day 21 when compared with the control group (p < 0.01; Fig. 1). On day 21, the mean tumor volume was 18.93 ± 5.39mm^3^ and 75.68 ± 25.83mm^3^ for melatonin-treated and control groups, respectively.

### DE genes by melatonin treatment were detected only in the TME

First, we aligned each RNA-Seq sample against two reference genomes, human (GRCh37/hg19) and mouse (NCBI37/mm9) separately. Data showed that 57.24% of reads mapped uniquely to human and 29.66% uniquely in mouse, while 11% of reads mapped to both human and mouse genomes and were excluded from further analyses (Supplementary Table S1).

**Figure 2:**
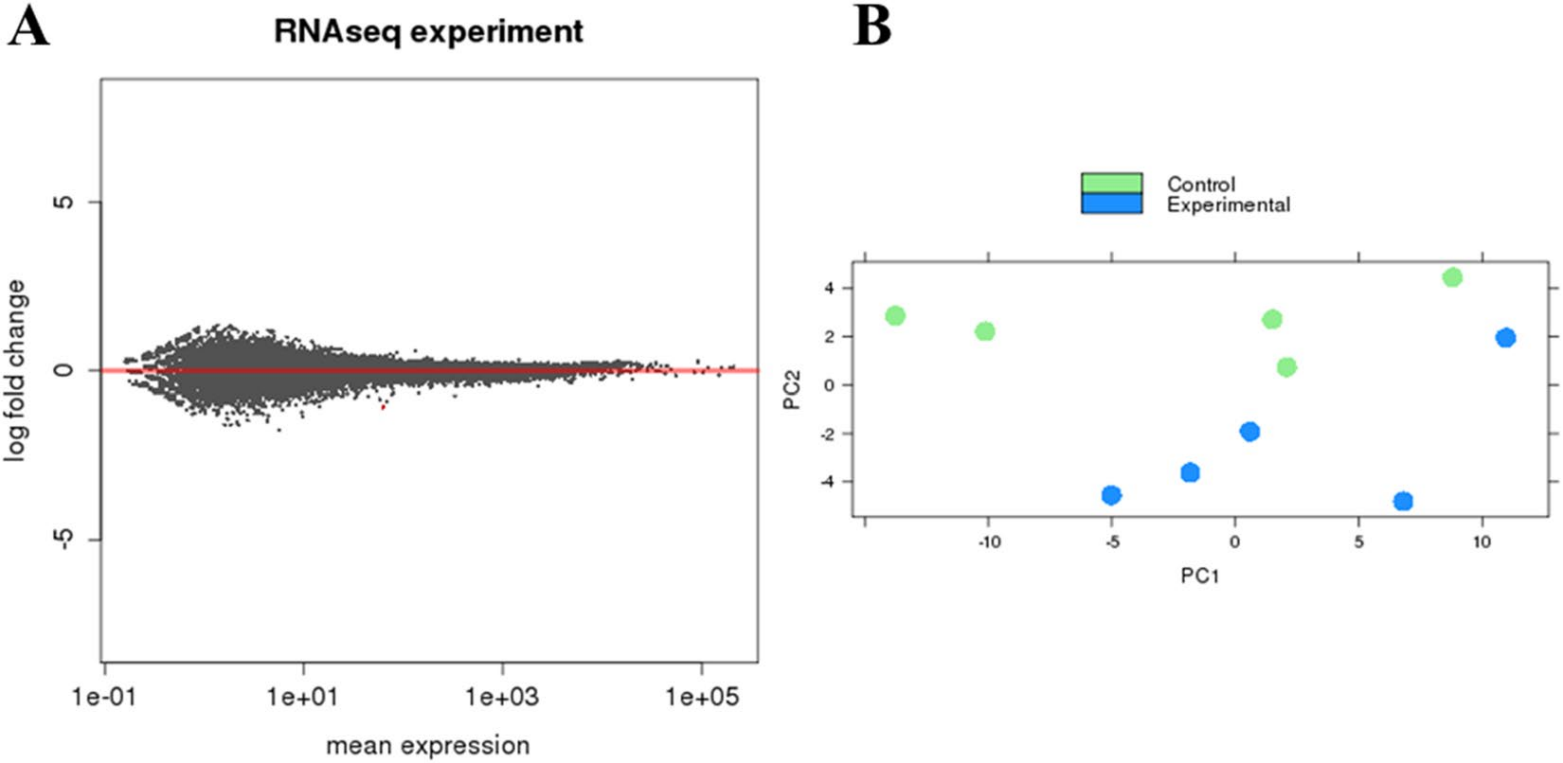
Differentially expressed (DE) genes between melatonin-treated and control groups in MDA-MB-231 human breast cancer cells. (**A**) Correlation analysis of gene expression changes between melatonin-treated samples vs. control samples. The log fold change is plotted on the y-axis and the mean expression of the reads counts is shown on the x-axis. Each point represents a gene. Red points indicate genes called as differentially expressed (DE) at adjusted p value (adjP) ≤ 0.1. (**B**) Principal component (PC) analysis was performed for the samples using the gene expression values. Experimental group in green represents the melatonin-treated samples. Control group in blue represents the vehicle-treated samples. Scatter plot model of differential gene expression ratios in melatonin-treated breast cancer (blue dots) vs. control (green dots) shows a reasoned discrimination where the distance between dots is a dimensional measure for the similarity of the expression profiles.

A total of 31807 genes were detected for the reads aligned to the human genome, that is, those from MDA-MB-231 cancer cells. Genes were tested for differential expression (Fig. 2), however, only the pseudogene GAPDHP65 was DE after melatonin treatment showing log2 FC -1.07 and padj = 0.01.

**Figure 3:**
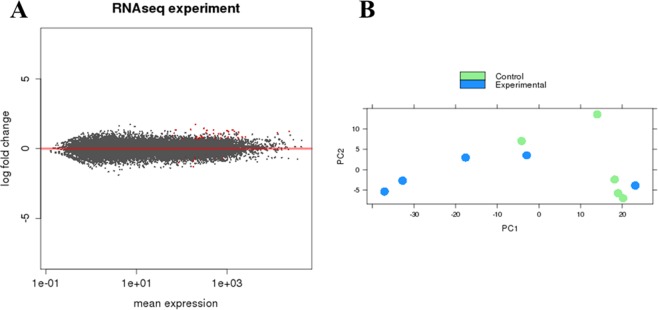
Differentially expressed (DE) genes between melatonin-treated and control groups in mouse cells, characterized as the tumor microenvironment (TME). (**A**) Correlation analysis of gene expression changes between melatonin-treated samples vs. control samples. The log fold change is plotted on the y-axis and the mean expression of the reads counts is shown on the x-axis. Each point represents a gene. Red points indicate genes called as differentially expressed (DE) at adjusted p value (adjP) ≤ 0.1. (**B**) Principal component (PC) analysis was performed for the samples using the gene expression values. Experimental group in green represents the melatonin-treated samples. Control group in blue represents the vehicle-treated samples. Scatter plot model of differential gene expression ratios in melatonin-treated breast cancer (blue dots) vs. control (green dots) shows a reasoned discrimination where the distance between dots is a dimensional measure for the similarity of the expression profiles.

In mouse cells, which represent the TME, 24719 genes were detected. Of those, 57 genes were DE between melatonin-treated and control groups (adjP < 0.1) (Fig. 3). Of those, 14 genes were downregulated (log2 FC ≤ −0.58) and 43 upregulated by melatonin treatment (lod2 FC ≥ 0.58) (Supplementary Table S2).

The functional enrichment analysis of GO terms showed that the DE genes of TME were mainly involved in immune system processes and cell motility, such as inflammatory response, regulation of cell motility and leukocyte migration, regulation of chemotaxis and chemokine production (Table 1). In addition, pathways enriched for DE genes in TME after melatonin treatment included the chemokine signaling, pathways in cancer and Wnt signaling, as shown in Table 2. These results elucidated a potential role of melatonin in the immune response in the TME.

**Table 1:** DE genes associated with the most relevant biological processes enriched for the TME (mouse tissue) after melatonin treatment. Last column shows the biological processes associated with each gene, represented by numbers and detailed bellow.

Gene	Log2FC	Gene name	Entrez Gene	Numbers corresponding to biological processes
F2rl1	−0.75	coagulation factor II (thrombin) receptor-like 1	14063	1-2-3-4-5-6-7-9-10-11-13-14
Dsg2	−0.95	desmoglein 2	13511	1-16
Egfl7	0.68	EGF-like domain 7	353156	1-3-4-5
Aif1	0.76	allograft inflammatory factor 1	11629	1-2-3-4-5-6-7-8-9-10-16
Ccl12	0.83	chemokine (C-C motif) ligand 12	20293	1-2-6-7-8-9-12-13-14
Mmp3	0.80	matrix metallopeptidase 3	17392	1-3-4-5-16
Apod	0.63	apolipoprotein D	11815	1-2-3-4-5-6-11-12-13-14-15
Prkca	−0.66	protein kinase C, alpha	18750	1-2-3-4-5-6-7-9-10-12-13-14-15
Mdk	0.92	Midkine	17242	1-3-4-5-14
**Tnfaip8l2**	0.79	tumor necrosis factor, α-induced protein 8-like 2	69769	2-12-13-14
Ctla2a	0.72	cytotoxic T lymphocyte-associated protein 2 α	13024	2-13-14
Ifit3	0.76	interferon-induced protein with tetratricopeptide repeats 3	15959	2-14
Ereg	0.78	Epiregulin	13874	2-14
Krt1	1.23	keratin 1	16678	2-14
**Il1f6**	1.74	interleukin 1 family, member 6	54448	13-14
Penk	−0.59	Preproenkephalin	18619	14
Defb14	1.33	defensin beta 14	244332	14
Gpx3	0.82	glutathione peroxidase 3	14778	15

Biological process numbers are: (1) Cellular component movement (ID:GO:0006928); (2) Immune system process (ID:GO:0002376); (3) Regulation of cell motility (ID:GO:2000145); (4) Regulation of cell migration (ID:GO:0030334); (5) Cell migration (ID:GO:0016477); (6) Leukocyte migration (ID:GO:0050900); (7) Leukocyte chemotaxis (ID:GO:0030595); (8) Monocyte chemotaxis (ID:GO:0002548); (9) Cell chemotaxis (ID:GO:0060326); (10) Positive regulation of chemotaxis (ID:GO:0050921); (11) Negative regulation of chemokine production (ID:GO:0032682); (12) Regulation of inflammatory response (ID:GO:0050727); (13) Inflammatory response (ID:GO:0006954); (14) Defense response (ID:GO:0006952); (15) Response to reactive oxygen species (ID:GO:0000302).

**Table 2:** Pathways enriched for differently expressed (DE) genes in tumor microenvironment (TME) (mouse tissue) after melatonin treatment.

PathwayName	Genes	Statistics
Chemokine signaling pathway	Ccl12, Gng11, Gngt2	C = 185; O = 3; E = 0.18; R = 17.06; rawP = 0.0007; adjP = 0.0042
Phosphatidylinositol signaling system	Prkca, Calm4	C = 78; O = 2; E = 0.07; R = 26.97; rawP = 0.0025; adjP = 0.0058
Glioma	Prkca, Calm4	C = 66; O = 2; E = 0.06; R = 31.88; rawP = 0.0018; adjP = 0.0058
ErbB signaling pathway	Prkca, Ereg	C = 87; O = 2; E = 0.08; R = 24.18; rawP = 0.0032; adjP = 0.0067
Pathways in cancer	Fzd1, Prkca, Col4a5	C = 325; O = 3; E = 0.31; R = 9.71; rawP = 0.0037; adjP = 0.0071
GnRH signaling pathway	Prkca, Calm4	C = 99; O = 2; E = 0.09; R = 21.25; rawP = 0.0041; adjP = 0.0072
Leukocyte transendothelial migration	Prkca, Cldn5	C = 120; O = 2; E = 0.11; R = 17.53; rawP = 0.0059; adjP = 0.0087
Vascular smooth muscle contraction	Prkca,Calm4	C = 123; O = 2; E = 0.12; R = 17.11; rawP = 0.0062; adjP = 0.0087
Tight junction	Prkca, Cldn5	C = 137; O = 2; E = 0.13; R = 15.36; rawP = 0.0076; adjP = 0.0094
Insulin signaling pathway	Ppp1r3b, Calm4	C = 137; O = 2; E = 0.13; R = 15.36; rawP = 0.0076; adjP = 0.0094
Cell adhesion molecules (CAMs)	Nectin3, Cldn5	C = 149; O = 2; E = 0.14; R = 14.12; rawP = 0.0089; adjP = 0.0104
Wnt signaling pathway	Fzd1, Prkca	C = 154; O = 2; E = 0.15; R = 13.66; rawP = 0.0095; adjP = 0.0105
Calcium signaling pathway	Prkca, Calm4	C = 178; O = 2; E = 0.17; R = 11.82; rawP = 0.0126; adjP = 0.0132
Focal adhesion	Prkca, Col4a5	C = 200; O = 2; E = 0.19; R = 10.52; rawP = 0.0157; adjP = 0.0157

C = the number of reference genes in the category; O = the number of genes in the gene set and also in the category; E = the expected number in the category; R: ratio of enrichment; rawP = p value from hypergeometric test; adjP = p value adjusted by the multiple test adjustment.

### Co-expression analysis between control and melatonin-treated groups

The aim of differential connectivity analysis was to uncover differences in gene behavior in two sub-networks, one created by expression data from 5 control tumors and another created by expression data from 5 melatonin-treated tumors. Human and mouse data were analyzed separately.

In tumor cells, 714 differentially co-expressed genes were detected between melatonin-treated and control groups (K_diff_ > |0.6|). Of those, 331 were highly connected in control tumors, while 383 were highly connected in melatonin-treated tumors (Supplementary Table S3). Functional enrichment analysis of GO terms for the highly connected genes in melatonin-treated tumors is shown in Supplementary Fig. S1. For the control tumors, the 331 connected genes were enriched to cytokine binding and cellular component major histocompatibility complex (MHC) protein complex, suggesting an association with the immune system (Supplementary Fig. S2). Interestingly these connected genes from control tumors were also enriched for signaling pathways related with immune response, such as “Antigen processing and presentation”, “Allograft rejection” and “Acute graft-versus-host disease”, as well as the cell adhesion molecules (CAMs) pathway, which plays a critical role in the immune response (Supplementary Table S4).

For the TME, 13899 genes passed the quality control for the co-expression analysis. 1345 genes were identified as differentially co-expressed in the TME between melatonin-treated and control groups. Of those, 1047 were highly connected in the control group, while 298 were highly connected in melatonin-treated tumors (Supplementary Table S5).

Highly connected genes in the melatonin-treated group were enriched to processes related with the inhibition of cellular processes, such as “negative regulation of cellular process” and “negative regulation of RNA metabolic process” (Supplementary Fig. S3). Interestingly, the opposite was observed for the 1047 connected genes in the control group. Most of the biological processes enriched for the control group were those negatively regulated in the melatonin-treated group, suggesting that melatonin treatment is counteracting the pro-tumor cellular processes (Supplementary Fig. S4). In addition, the “protein processing in endoplasmic reticulum” pathway was also enriched in the control group, suggesting high cellular activity, consistent with cell proliferation and tumor growth.

### Module preservation analyses between control and melatonin-treated groups

**Figure 4:**
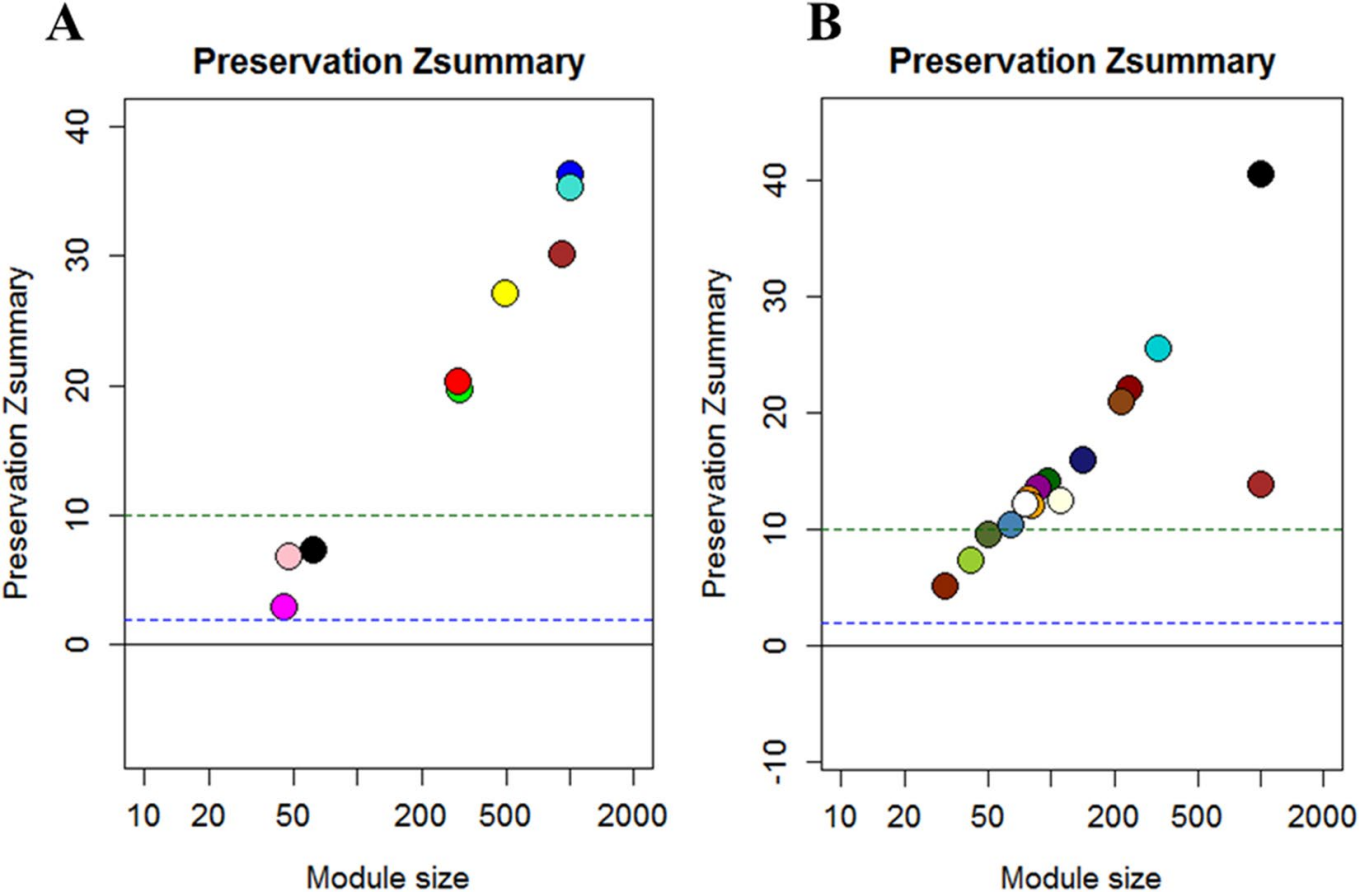
Weighted gene coexpression network analysis (WGCNA) identified modules of co-expressed genes. Values of Zsummary above 10 represent modules of genes highly preserved. Values between 2 and 10 represent poor to moderate preservation, which can indicate sets of genes influenced by melatonin treatment. (**A**) Three modules of genes with moderated preservation (pink, magenta and black) were identified to MDA-MB-231 human breast cancer cells; (**B**) Three modules of genes with moderated preservation (darkolivegreen, yellowgreen and orangered) were identified to the tumor microenvironment (murine cells).

Gene members of the same module are supposed to work cooperatively in related pathways or be under the control of a common set of transcription factors. Thus, 10 gene modules of co-expressed and highly interconnected genes were identified in human tumor cells. Of those, three modules (pink, magenta, and black) were discovered to show weak to moderate preservation, which may indicate that melatonin is acting on these gene sets (Fig. 4A). The pink module was composed of 48 genes, magenta was composed of 45 genes and black was composed of 62 genes (Supplementary Table S6). Genes of each individual module were tested for functional enrichment analyses. Genes from the black module showed enrichment for the cellular component nuclear matrix (Supplementary Fig. S5A), while genes from magenta module were enriched to intracellular-membrane organelle (Supplementary Fig. S5B) and 5 genes were involved in the metabolic pathway.

In the TME cells, 39 modules were found and because of the high number of modules identified, they were grouped using a threshold of 90% correlation between the expression of the modules, resulting in a total of 16 co-expressed gene modules. Of those, three modules (darkolivegreen, yellowgreen and orangered) were strongly associated with melatonin treatment in the TME (Fig. 4B), as they showed weak to moderate preservation. These modules were composed of 49, 41 and 30 genes for darkolivegreen, yellowgreen and orangered, respectively (Supplementary Table S7).

Genes from the darkolivegreen module were enriched to processes related to posttranscriptional regulation of gene expression (Supplementary Fig. S6), while those from yellowgreen module were enriched to “Positive regulation of cell cycle arrest”, “Cell cycle checkpoint” (Supplementary Fig. S7) and the TGF-beta signaling pathway, which has a wide spectrum of cellular functions such as apoptosis, cell proliferation, differentiation, and migration. Genes from orangered module were related to immune system development, leukocyte differentiation, T cell differentiation, and the signaling pathways of Focal adhesion and Regulation of actin cytoskeleton (Supplementary Fig. S8). These data again suggest melatonin’s action is in regulating the immune system in the TME.

### Validation of genes modulated by melatonin treatment

**Figure 5:**
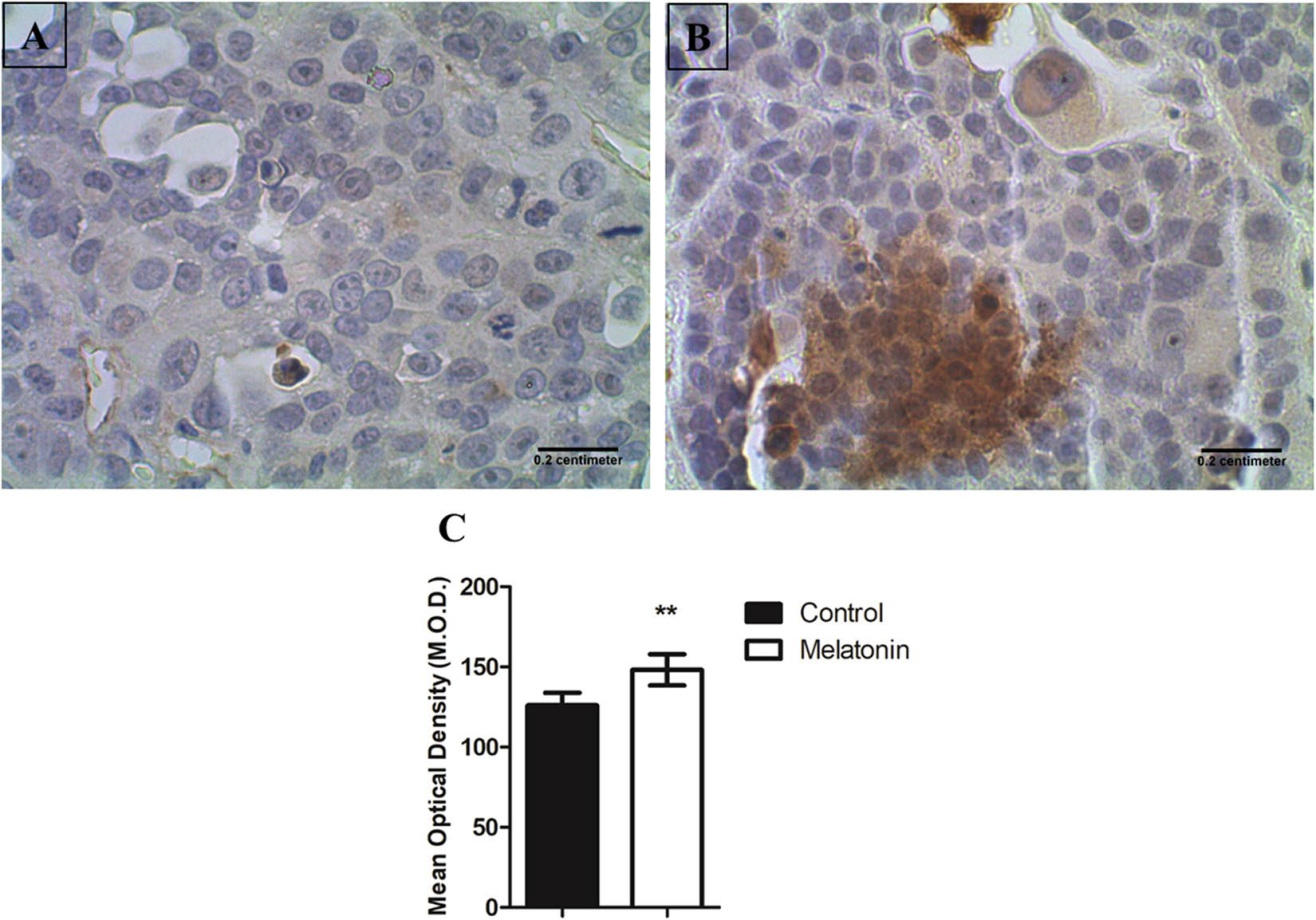
Immunohistochemistry staining with Cleaved Caspase-3 in breast cancer cells. (**A**) Representative image of control and (**B**) melatonin-treated tumors. Images were taken with 40X magnification. Scale bar = 0.2 cm. (**C**) A significant increase was observed in melatonin-treated tumors compared with control tumors (**p = 0.0078). Image analysis was performed by optical density to quantify the relative intensity of immunoreactivity. Values were obtained as arbitrary units (a.u.) and the mean optical density values were compared between groups using Student’s t-test.

Melatonin does not appear to have its principal activity at the gene level in tumor cells, as there was no DE gene in the melatonin-treated tumor cells. Co-expression and module preservation analyses have shown processes related to cell proliferation and tumor growth in melatonin treated-tumors, which are consistent with the reduced tumor growth, and most likely with an increase of apoptosis in melatonin-treated tumors. Thus, to validate these findings we evaluated the expression of cleaved caspase-3 at protein levels in the tumor tissue by immunohistochemistry. In fact, results showed that cleaved caspase-3 expression was higher in melatonin-treated tumors, indicating the pro-apoptotic effect of melatonin (Fig. 5).

**Figure 6:**
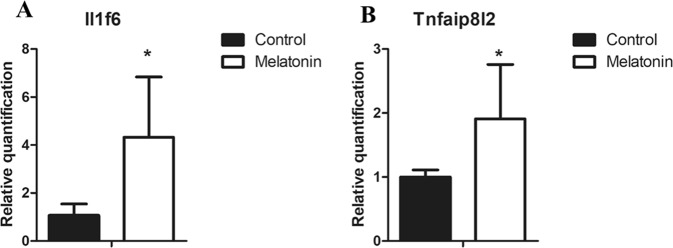
Gene expression analysis by Real time PCR in the tumor microenvironment (TME). (**A**)Relative quantification of Il1f6 gene expression; (**B**) Relative quantification of Tnfaip8l2 gene expression. Genes were normalized to gapdh expression. Values are expressed as mean ± standard deviation (SD) of experiments performed in triplicate. *p < 0.05 melatonin-treated tumor vs. control tumors by Student t-test.

For the TME, composed of murine cells, DE and co-expression analyses showed that melatonin influences several processes related to the immune system. Thus, we selected two DE genes affected by melatonin treatment to perform individual validation by real-time PCR. Interleukin 1 family, member 6 (Il1f6), also known as Interleukin-36α (IL-36α) and Tumor necrosis factor-a (TNF-a)-induced protein 8-like-2 (Tnfaip8l2) were selected to validate, as these genes are involved in the immune response and therefore may play an important role in the TME. Both genes were upregulated by melatonin treatment as evidenced by RNA-Seq results and its increase was confirmed by real-time PCR. Expression of Il1f6 and Tnfaip8l2 genes were significantly increased in melatonin-treated tumors when compared with control tumors (Fig. 6).

## Discussion

Melatonin has a broad spectrum of actions ranging from the regulation of physiological functions, such as the circadian rhythm and redox state, to anti-cancer functions that control the development and growth of different types of tumors^39,40^. Its anti-proliferative and pro-apoptotic effects have been described in a variety of tumors in both *in vitro* and *in vivo* studies^17,18,41^. In this study, we showed that melatonin treatment reduced the tumor growth in a xenograft breast cancer model, and consistent with its reduction, melatonin was able to increase the protein expression of cleaved caspase-3, which is the main inducer of apoptosis. Similarly, in a DMBA-induced mammary carcinoma rat model, melatonin treatment increased apoptosis as observed by the upregulation of tissue caspase-3 activity, TNF-alpha and the percentage of DNA fragmentation^41,42^. In addition, in a previous study, we showed that melatonin also acted as an anti-proliferative agent, reducing ki-67 protein expression and consequently the tumor growth^11^. In the current study, we reconfirmed melatonin reduces tumor growth and we performed a global analysis by RNA-Seq to identify the melatonin actions not only in tumor cells but also in the TME.

It is known that melatonin is a pleiotropic anti-cancer molecule^43^. Recently, Reiter *et al*. described it as a ubiquitously-distributed molecule and suggested that its diverse actions could be mere epiphenomena of an underlying more fundamental melatonin action that remains to be discovered^18^. In our study, RNA-Seq analysis showed that melatonin treatment did not change gene expression directly in the tumor cells, but it modulates gene expression in the TME, which may have contributed in controlling the tumor growth. DE and co-expressed genes, as well as the gene modules that were influenced by melatonin treatment, showed functional enrichment mainly to processes associated with different aspects of immune response.

It has been shown that immune cells express both membrane and nuclear melatonin receptors^41,44^. In addition, these cells have the enzymes to produce melatonin^45^, which can act as a paracrine, intracrine or an autocrine agent^41,44,46^. Thus, melatonin may influence immune responses in different levels^47^.

Specifically, we showed that DE genes affected by melatonin treatment in the TME were mostly associated with cellular component movement and immune system processes. To note, this is the first study that evaluated the action of melatonin by RNA-Seq in a xenograft model. Few studies have used RNA-Seq to evaluate the action of melatonin *in vitro*. Through an RNA-Seq experiment, Mori *et al*.^48^ identified 14 upregulated genes following melatonin treatment in HCT116 colon cancer cells. Four of these 14 genes (p53, p38, PML, and H2AX) were described as miR-24–3p putative targets, an oncogenic microRNA. Melatonin decreased the miR-24 levels in HCT116 and MCF-7 cell lines, therefore reducing several pro-tumor processes associated with its target genes, such as cell proliferation, migration, DNA damage, cell transformation, and RNA metabolism, evidencing that miR-24 downregulation could be a major event among the anti-tumor actions of melatonin. Other studies using RNA-Seq experiments identified different mechanism of actions for melatonin, which involved inhibition of cell motility, invasion and migration in hepatocarcinoma^49^ and osteosarcoma cell lines^50^. In the breast cancer cell line MCF-7 treated with melatonin (1 nM and 100 nM for 72 hours), a transcriptomic analysis was performed using microarray gene expression profiling. Authors identified a large number of DE genes following melatonin treatments (1946 upregulated and 983 downregulated). As expected, those genes were mainly involved in cell proliferation, adhesion, apoptosis, cell cycle, as well as immune response^51^.

In this study, in addition to the differential expression analysis to identify DE genes, we performed differential connectivity and module preservation analyses. These results were complementary to the DE genes and confirmed the action of melatonin mainly on the immune system. First, the differential connectivity analysis showed that connected genes in the tumor cells from the control group were associated with signaling pathways that indicate an immune response in a xenograft model, such as allograft rejection, acute graft-versus-host disease, and antigen processing and presentation. These results may indicate the processes that are occurring in the tumor cells to establish and develop a tumor in the murine host environment. Although we used a T-cell deficient mouse model (athymic Foxn1nu) that has limitations to studying the response to therapy^52^, it is important to note that these mice show an intact humoral adaptive immune system as well as an intact innate immune system^53^, which could, to some extent, lead to an immune response.

On the other hand, connected genes in the TME from the control group were associated with positive regulation of cellular processes consistent with cell proliferation and tumor growth. Interestingly in the melatonin-treated TME, connected genes were associated with negative regulation of those processes, suggesting that melatonin treatment is inhibiting pro-tumor cellular processes. In the module preservation analyses, 3 gene modules were identified as being strongly influenced by melatonin treatment in the TME, which were associated with cellular processes involved in cell proliferation and again in the regulation of immune system, evidencing once again the key role of melatonin in the immune response in the TME.

According to our results, melatonin’s effects on the immune system have been described in several studies. Melatonin showed immunoenhancing properties^54^, and its exogenous administration enhanced both innate and cellular immunity^41^, being able to regulate inflammatory cytokines and mediators, expression of transcription factors genes^47^, and the haemopoiesis^55^. Melatonin administration also enhanced the production of progenitor cells for granulocytes-macrophages, natural killer cells, monocytes, and leukocytes^41^, as well as the production of cytokines including interleukins (IL-2, IL-6, IL-12), interferon-gamma and TNF-alpha^54,56^. Although many studies have implicated melatonin as a positive regulator of the immune system, others have described melatonin as an anti-inflammatory^45^. In the early phases of inflammation, melatonin can activate pro-inflammatory mediators, such as IL-1 and TNF-alpha, while in the chronic phase, it contributes to the inflammation attenuation by downregulating cytokines, inducing the survival pathway in leukocytes and blocking oxidative stress by its antioxidant properties^41,45,47,57^. In our results, melatonin treatment was also able to upregulate the glutathione peroxidase 3 (Gpx3) in the TME, an important antioxidant enzyme.

In this context, the effects of melatonin on the immune system were observed in our study, which may modulate the inflammatory response by different mechanisms. Specifically, we validated melatonin action on two genes involved in the immune response and tumor progression. Il1f6 shows an important pro-inflammatory role in chronic immune disorders^58,59^ and recently its role in cancer has been described^59,60^. This gene is a member of the IL-1 family of cytokines, also composed of two other agonists, IL-36-β (IL-1F8) and IL-36γ (IL-1F9) and one antagonist IL-36Ra (IL-1F5)^61,62^. The IL-36 family of cytokines supports the generation of pro-inflammatory immune responses and its possible functions in cancer still under investigation^62^. In 2014, Wang *et al*. reported that low expression of IL-36α was correlated with larger tumor size and poor prognosis of colorectal cancer patients^63^.

Recent studies have demonstrated the use of IL-36 as an immunotherapeutic agent in cancer therapy. Chang *et al*. showed that IL-36 gene therapy can inhibit the growth in xenograft model of epithelial ovarian cancer^59^. Moreover, its upregulation was able to regress the tumor masses in fibrosarcoma mouse model and reduce the Ki-67 expression^60^. IL-36γ exerted profound antitumor effects in a melanoma mice model, transforming the TME in favor of tumor eradication^62^. An interesting review by Weinstein and Storkus describes IL-36 as an orchestrator of Tertiary Lymphoid Structures (TLS), an ectopic lymphoid formation, in the TME. They suggest that IL-36 production in the TME has potential protective and therapeutic roles in a cancer-bearing host, by acting in both humoral and cellular immunity^64^.

Another gene involved in the immune system and upregulated by melatonin in the TME was the Tnfaip8l2, which is a member of the tumor necrosis TNFAIP8 family and an essential negative regulator of both innate and adaptive immunity, showing high expression in immune cells^65^. Despite its role in maintaining immune homeostasis, Tnfaip8l2 is also involved in the development and progression of several tumors^66,67^. Its expression is reduced or absent in some cancers, such as gastric cancer, lung cancer, hepatocellular carcinoma^66,68^, and breast cancer^65^. Recently, Wang *et al*. showed that the overexpression of Tnfaip8l2 inhibited the tumor growth in a xenograft MDA-MB-231 breast cancer model. In addition, Tnfaip8l2 prevented the EMT phenotype, by inhibiting the expression of β-catenin, cyclin D1 and c-Myc in MDA-MB-231 and MCF-7 breast cancer cells^69^. Similar results were also reported in a xenograft model of gastric cancer. Adenovirus-mediated Tnfaip8l2 overexpression suppressed the tumor growth by increasing apoptosis and reduced cell migration, invasion and metastasis via reversal of EMT^70^.

Zhang *et al*., using a syngeneic model of breast cancer (Balb/c mice implanted with 4T1 cells) showed that the overexpression of Tnfaip8l2 inhibited the proliferation of 4T1 cells *in vitro* and *in vivo*. Interestingly, the treatment with Tnfaip8l2 gene delivery *in vivo* promoted CD8 + T and NK cell-mediated anti-tumor immune responses in the TME. In addition, Tnfaip8l2 inhibited the expansion and recruitment of myeloid-derived suppressor cells (MDSCs), which exert immune-suppressive effects and promote tumor progression^71^. Previously, they showed that the overexpression of Tnfaip8l2 in MDA-MB-231 cells leads to a reduction of proliferation, migration, and invasion *in vitro* and inhibits the tumorigenesis of breast cancer *in vivo*^65^.

Taken together, studies indicate the importance of Tnfaip8l2, as well as the Il1f6, on the immune response. Thus, the capacity of melatonin to increase these genes in the TME suggests additional actions of melatonin that contribute to the control of tumor growth.

In conclusion, our study has shown that melatonin acts by regulating gene expression mainly in the TME and suggest new actions to its molecule on modulation of immune response and tumor growth control in an MDA-MB-231 breast cancer xenograft mouse model. It is important to mention the limitations of this study, which analyzed the melatonin actions only in one pre-clinical tumor model, therefore limiting our conclusions to this specific type of cancer. However, our results from the transcriptomic analysis open possibilities to focus on specific targets in triple negative breast cancer patients, in order to understand the effects of melatonin in this type of tumor, that lacks specific therapeutic targets. Moreover, the current results merit further investigations in other tumor types, and more importantly, show an important action of melatonin in the TME, which is a field worth exploring in future investigations.

## Methods

### Breast cancer xenograft model

All procedures were approved by the Ethics Committee on the Use of Animals of Faculdade de Medicina de São José do Rio Preto (001-003336/2014) and developed following national and international standards of ethics in animal experimentation.

Human triple-negative breast cancer cell line MDA-MB-231 (American Type Culture Collection, Manassas, Virginia, USA) was cultured in RPMI-1640 (Life Technologies, Carlsbad, California, US) with 10% of fetal bovine serum (FBS) (Life Technologies, Carlsbad, California, US) and 1% of penicillin/streptomycin (Life Technologies, Carlsbad, California, US) at 37 °C and 5% CO_2_. Cells were detached using trypsin, suspended in the growth medium, washed with phosphate-buffered saline (PBS) twice and re-suspended at the 3 × 10^6^ cells to be inoculated in the right 4 th mammary gland of Balb/c athymic nude mice (n = 10).

Mice were randomly divided in control and melatonin-treated groups. The treatment was performed in accordance with a previous study^11^. Mice received melatonin (Sigma, St. Louise, MO, USA) (100 µl at the dose of 40 mg/kg of body weight) by intraperitoneal injection (IP) 5 days a week, while control mice received IP injection with 100 µl of vehicle solution (8 phosphate buffered saline (PBS): 1 dimethyl sulfoxide (DMSO): 1 Cremophor (Sigma, St. Louise, MO, USA). Treatments were given 1 hour before room lighting was switched off. Mouse weight was periodically monitored during the experiment. Tumors were measured by digital caliper and tumor volume was calculated as 1/2 (length × width^2^).

### Samples collection and RNA extraction

Mice were euthanized using ketamine and xylazine, tumors were collected and immediately frozen in liquid nitrogen. Total RNA was extracted using TRIZOL reagent (Invitrogen Life Technologies®, Sao Paulo, SP, BR). Tumors were homogenized using 1 ml of TRIZOL per 50 mg of tissue. 200 µl of chloroform were added per 1 mL of Trizol for each sample. Samples were incubated for 3 minutes in room temperature and centrifugated for 15 minutes at 12000 × g at 4 °C. The aqueous phase containing the RNA was transferred to a new tube and 500 µl of isopropanol was added. Solution was incubated for 10 minutes and centrifuged for 10 minutes at 12,000 × g at 4 °C. Following, the supernatant was discarded and the pellet containing the RNA was resuspend in 1 mL of 75% ethanol. Solution was centrifuged for 5 minutes at 7500 × g at 4 °C and the supernatant was discarded. The RNA pellet was left to dry for 10 minutes and resuspend in 20 µL of RNase-free water.

RNA quality and quantity were assessed using automated capillary gel electrophoresis on Bioanalyzer 2100 with RNA 6000 Nano Labchips (Agilent Technologies Ireland, Dublin, Ireland). First, the reagents were left to equilibrate in room temperature for 30 minutes. Then, 1 μl of RNA 6000 Nano dye concentrate was added to 65 μl of Agilent RNA 6000 Nano gel matrix. This solution was vortexed thoroughly to proper mixing of gel and dye and centrifuged for 10 minutes at room temperature at 13000 g. Gel-Dye was loaded into a new RNA chip and then 1 µl of RNA 6000 Nano Marker and 1 µl of each sample were added into the wells. The chip was vortexed for 1 minute at 2400 rpm and inserted in the Agilent 2100 Bioanalyzer to run the analysis. All samples presented RNA integrity number (RIN) higher than 8.0 (Supplementary Fig. S9).

### Preparation and Sequencing of Illumina RNA libraries

RNA-Seq libraries were created using Truseq RNA-Seq Library Prep Kit-v2. The experiment was paired-end with 100nt read length, performed on the Illumina HiSeq. 2500 sequencer.

In summary, 1 μg of total RNA was used to isolate mRNA poli(A) by two rounds of purification using oligo dT magnetic beads followed by fragmentation and cDNA synthesis by random hexamer primers and reverse transcriptase. Next, end repair and 3′ ends adenylation of the fragments was performed by adding a nucleotide A (*A-Tailing Mix)* to the 3′ end in order to prevent them to binding to each other during the ligation of adapters. Bar-coded adapters were ligated to the cDNA fragments and a PCR reaction was performed to produce the sequencing libraries. The quality of libraries and quantification were performed using Agilent 2100 Bioanalyzer and qPCR with KAPA Library Quantification kit (KAPA Biosystems, Foster City, USA). Adapter-ligated cDNA fragment libraries were run on Illumina HiSeq. 2500 equipment using TruSeq PE Cluster Kit and TruSeq SBS Kit (2 × 100 bp). All 10 samples were sequenced in one lane, producing about 30 million reads per library.

### Reads alignment and differential expression analysis

Sequencing quality was evaluated by FastQC software (http://www.bioinformatics.babraham.ac.uk/projects/fastqc/). Adaptors and poor quality bases were trimmed by Trimmomatic software^72^ and only reads with at least 50 bp in length were kept for further analyses.

An alignment strategy was used to especially analyze data of xenograft samples, which contain two genomes. Therefore, to differentiate human and mouse expression we used a strategy similar to the species-specific RNA-Seq workflow used by Bradford *et al*.^33^. Common reads between the two genomes (those aligning with equal similarity in human and in mouse) were discarded. Although some information may be lost by discarding the “common reads”, it is an important step to avoid false positive.

To differentiate human and mouse expression, the alignment was performed to filter out mouse-like reads before mapping to the human reference and vice versa, and data were mapped against human (GRCh37/hg19) and mouse (NCBI37/mm9) genomes separately, using the STAR software. The HTSeq was employed for read counts and DESeq. 2 was used to identify DE genes between melatonin treated and control tumors for each species. DE genes were obtained for each organism: human characterizing the MDA-MB-231 tumor cells and mouse characterizing the TME. Genes with adjusted p-value (adjP) ≤ 0.1 and fold change (FC) values ≤ −1.5 and ≥1.5 were considered as DE genes and down-regulated or up-regulated in the melatonin-treated group, respectively.

### Differential connectivity analysis

As a quality control, genes with zero expression values in more than half (5) of the samples and genes with average read counts less than 10 and standard deviation less than 5 were excluded. Genes with low counts may represent a bias of sequencing and genes with little variation contribute less to the network analysis. A total of 13669 human genes and 13899 mouse genes remained. Data were then divided into 2 sub-networks, one composed of melatonin-treated mice and the other composed of control mice. The sub-networks were created using WGCNA package in R environment^73^, and the total connectivity value of each gene within the network was calculated. Connectivity is the sum of the correlations between the expression of a given gene and the expression of each of the other genes in the network. Connectivity values of each gene in the two sub-networks were divided by the maximum connectivity found in each sub-network, so that the two sub-networks could have comparable values. The connectivity value of each gene from the control group was then subtracted from the connectivity value of the same gene in the melatonin-treated group. The resulting values ranged from 1 to −1 and genes with values greater than |0.6| were considered differentially connected. Negative values are associated with highly connected genes in the control group and poorly connected in the melatonin-treated group, while positive differential connectivity values are associated with highly connected genes in the melatonin-treated group and poorly connected in the control group. A change in gene behavior, from highly connected to lowly connected and vice-versa can indicate a response to melatonin treatment.

### Module preservation analysis

The same 13669 human genes and 13899 mouse genes which passed quality control were used to create a smaller set of genes, by selecting the 3000 most connected genes in each of the groups (melatonin vs control), which resulted in a total of 4557 human genes and 5383 mouse genes. A gene co-expression analysis was then performed using WGCNA package in R environment and modules of co-expressed genes were identified for the control group. Different color names were assigned to each module. Those modules were compared with the expression data of the melatonin-treated group for the evaluation of module preservation between the two groups. The preservation measurement is given by Zsummary were: modules with values greater than 10 are highly preserved; modules with values between 2 and 10 have poor to moderate preservation and modules with values below 2 are not preserved. Modules that are not preserved can indicate sets of genes influenced by melatonin treatment.

### Functional enrichment analysis

Functional enrichment of GO terms and KEEG analyses were performed using the WEB-based GEne SeT AnaLysis Toolkit (Webgestalt). Human and murine data were tested separately. For the DE gene lists, the specific genome for each organism was used as background, while a list of all genes that passed the quality control was used as background to each organism for the differentially connected genes and gene modules. P-values for each term were obtained through hypergeometric analysis and corrected for FDR by Benjamini–Hochberg method. Terms were considered significant when adjP ≤ 0.1.

### Real time PCR

Quantitative real-time PCR was used to validate the selected DE genes by melatonin treatment in the TME. Total RNA (100 ng) was used to generate cDNA by using the High-Capacity cDNA Reverse Transcription (Applied Biosystems). Each reaction included 20 ul of 2X RT master mix, containing 10X RT Buffer, 25X dNTP mix, 10X RT random primers, MultiScribe Reverse Transcriptase, nuclease-free water and 100 ng of RNA samples. Samples were incubated for 10 minutes at 25 °C, 120 minutes at 37 °C, 5 minutes at 85 °C and then stored at 4 °C. Gene expression of tnfaip8l2 and il1f6 was detected by real-time PCR (*Step One Plus* (*Applied Biosystems*) using SYBR Green master mix (Life Technology). Gapdh was used as the internal control. Primers sequences used were: *Tnfaip8l2* 5′-CTGGCTCTGGCTACACGATT-3′ (forward) and 5′-ACCTCACCGAAGCTAAGTGC-3′ (reverse); *Il1f6* 5′-CTCTTGAGACGAACAGGGGG-3′(forward) and 5′-ATGTTCCCTTCCCCAAGCTG-3′ (reverse); *Gapdh* 5′-GGTGAAGGTCGGTGTGAACG-3′(forward) and 5′-CTCGCTCCTGGAAGATGGTG-3′(reverse). Assays were run in 20 ul volume with final primer concentration of 200 nm for both reverse and forward primers. The conditions used were 2 minutes at 50 °C, 2 minutes at 95 °C, 40 cycles of 15 seconds at 95 °C followed by 15 seconds at 58 °C and 1 minute at 72 °C. Finally, a dissociation curve stage for 15 seconds at 95°, 15 seconds at 58 °C and 15 seconds at 95 °C. The relative expression of each gene was calculated using the 2^−ΔΔCt^. Experiments were performed in triplicate.

### Immunohistochemistry

Paraffin-embedded tissue sections of 3 µm were obtained, deparaffinized and rehydrated. Sections were incubated with Hydrogen Peroxide Block (Spring Bioscience, Pleasanton, CA, USA) for 10 minutes, washed in PBS 1×, incubated with Protein Block solution (Spring Bioscience, Pleasanton, CA, USA) for 10 minutes and washed again. Then, antigen retrieval was done at 95 °C with Citrate buffer (pH 6.0) for 30 minutes. Sections were incubated with anti-cleaved Caspase-3 antibody (Ab4051 - Abcam, Cambridge, MA, USA) at 4 °C overnight and then with horseradish peroxidase (HRP) conjugate, containing the goat anti-rabbit antibody conjugated to HRP, for 15 minutes at room temperature. Sections were washed and incubated with 3,3′-diaminobenzidine tetrahydrochloride (DAB) (Spring Bioscience, Pleasanton, CA, USA) for 3 minutes and then counterstained with Harris’s hematoxylin. Finally, sections were dehydrate and coverslipped with Permount mounting medium (Fisher Scientific,Hampton, New Hampshire, USA). Negative control was obtained by omitting the primary antibody, and tonsil was used as internal positive control of the assay.

Cleaved caspase-3 immunostaining was analyzed based on the intensity of the staining by optical density using ImageJ software (NIH, Bethesda MD, USA). Multiple fields from each histological slide were examined using the Nikon Eclipse E 200 microscope (Nikon Instruments, Melville, NY, USA). Then, three areas were photographed at 40X and 20 spots (small circular regions of interest) were randomly selected in each photographed area. Thus, the intensity was determined from a total of 60 spots of each sample to average the relative intensity of immunoreactivity. The values were obtained as arbitrary units (a.u.), and the mean optical density (M.O.D.) indicated the specific staining intensity in the immunoreactive areas.

### Statistical analyses

To analyze the real time PCR and immunohistochemistry data, the results were previously submitted to descriptive analysis to determine the normality. The mean values for each group (melatonin versus control) were compared by Student’s t-test. Analyses were performed using the GraphPad Prism6 software (Graph-Pad Software, La Jolla, CA, USA) and p-values less than 0.05 were considered statistically significant.

## Supplementary information

Supplementary figures

Supplementary Table S1

Supplementary Table S2

Supplementary Table S3

Supplementary Table S4

Supplementary Table S5

Supplementary Table S6

Supplementary Table S7

## Data Availability

All data generated or analyzed during this study are included in this published article and its Supplementary Information files.
