# Supplementary material for: RNA-Seq transcriptome analysis shows anti-tumor actions of melatonin in a breast cancer xenograft model
Source: Sci Rep. 2019 Jan 30;9:966. doi: 10.1038/s41598-018-37413-w (PMC6353949; doi:10.1038/s41598-018-37413-w)
Supplement: Supplementary file 1 — Supplementary figures [file 41598_2018_37413_MOESM1_ESM.pdf]

RNA-Seq transcriptome analysis shows anti-tumor actions of melatonin in a breast cancer xenograft model

Bruna Victorasso Jardim-Perassi; Pâmela A. Alexandre; Nathalia M. Sonehara; Rubens de Paula-Junior; Osvaldo Reis Júnior; Heidge Fukumasu; Roger Chammas; Luiz Lehmann Coutinho; Debora Aparecida Pires de Campos Zuccari

Supplementary figure legends

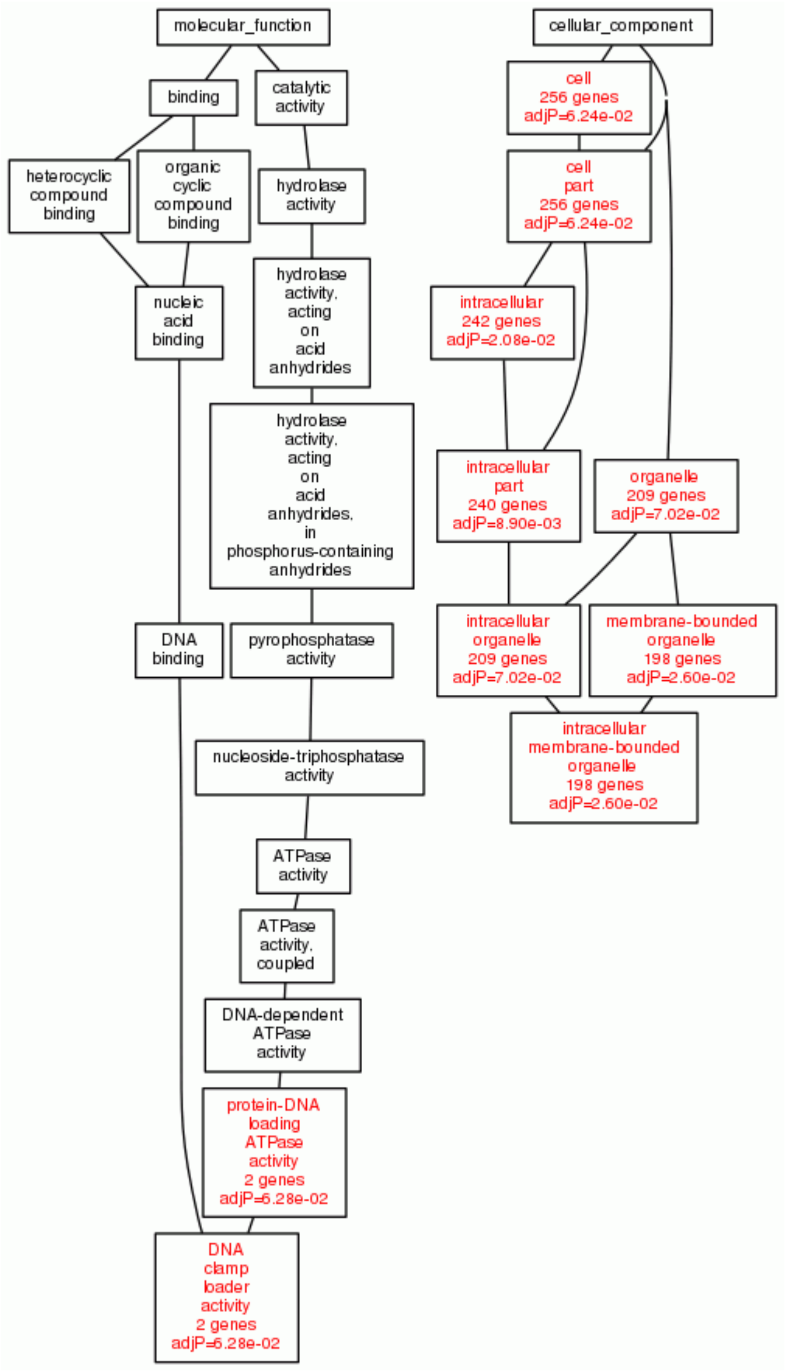

Supplementary figure S1: Gene ontology (GO) analysis of connected genes in the MDA-MB-21 human breast cancer cells of the melatonin-treated group. GO analysis was performed using Gene Set Analysis Toolkit software (www.bioinfo.vanderbilt.edu/webgestalt). The enriched GO categories are shown in red (hypergeometric; $P < 0.1$; red) and their nonenriched parents are shown in black.

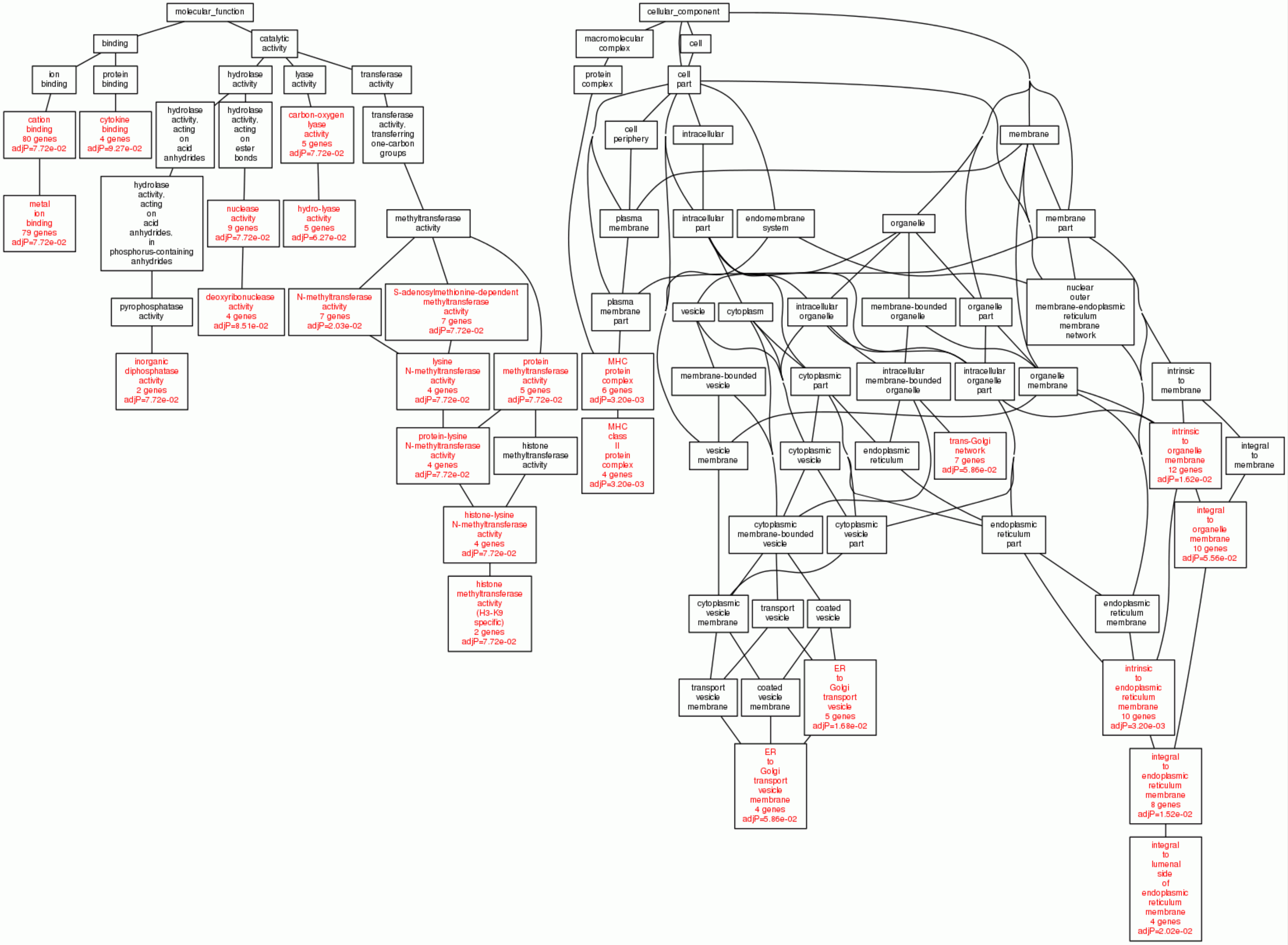

molecular_function
binding
catalytic activity
ion binding
protein binding
hydrolase activity
lyase activity
transferase activity
cation binding 80 genes adjP=7.72e-02
cytokine binding 4 genes adjP=9.27e-02
hydrolase activity, acting on acid anhydrides
hydrolase activity, acting on ester bonds
carbon-oxygen lyase activity 5 genes adjP=7.72e-02
transferase activity, transferring one-carbon groups
metal ion binding 79 genes adjP=7.72e-02
hydrolase activity, acting on acid anhydrides, in phosphorus-containing anhydrides
nuclease activity 9 genes adjP=7.72e-02
hydro-lyase activity 5 genes adjP=6.27e-02
methyltransferase activity
pyrophosphatase activity
deoxyribonuclease activity 4 genes adjP=8.51e-02
N-methyltransferase activity 7 genes adjP=2.03e-02
S-adenosylmethionine-dependent methyltransferase activity 7 genes adjP=7.72e-02
inorganic diphosphatase activity 2 genes adjP=7.72e-02
lysine N-methyltransferase activity 4 genes adjP=7.72e-02
protein methyltransferase activity 5 genes adjP=7.72e-02
protein-lysine N-methyltransferase activity 4 genes adjP=7.72e-02
histone methyltransferase activity
histone-lysine N-methyltransferase activity 4 genes adjP=7.72e-02
histone methyltransferase activity (H3-K9 specific) 2 genes adjP=7.72e-02
cellular_component
macromolecular complex
cell
protein complex
cell part
cell periphery
intracellular
membrane
plasma membrane
intracellular part
endomembrane system
organelle
membrane part
nuclear outer membrane-endoplasmic reticulum membrane network
plasma membrane part
vesicle
cytoplasm
intracellular organelle
membrane-bounded organelle
organelle part
intrinsic to membrane
MHC protein complex 6 genes adjP=3.20e-03
membrane-bounded vesicle
cytoplasmic part
intracellular membrane-bounded organelle
intracellular organelle part
organelle membrane
MHC class II protein complex 4 genes adjP=3.20e-03
vesicle membrane
cytoplasmic vesicle
endoplasmic reticulum
trans-Golgi network 7 genes adjP=5.86e-02
intrinsic to organelle membrane 12 genes adjP=1.62e-02
integral to membrane
cytoplasmic membrane-bounded vesicle
cytoplasmic vesicle part
endoplasmic reticulum part
integral to organelle membrane 10 genes adjP=5.56e-02
cytoplasmic vesicle membrane
transport vesicle
coated vesicle
endoplasmic reticulum membrane
transport vesicle membrane
coated vesicle membrane
ER to Golgi transport vesicle 5 genes adjP=1.68e-02
intrinsic to endoplasmic reticulum membrane 10 genes adjP=3.20e-03
ER to Golgi transport vesicle membrane 4 genes adjP=5.86e-02
integral to endoplasmic reticulum membrane 8 genes adjP=1.52e-02
integral to lumenal side of endoplasmic reticulum membrane 4 genes adjP=2.02e-02

Supplementary figure S2 caption: Gene ontology (GO) analysis of connected genes in the MDA-MB-21 human breast cancer cells of the control group. GO analysis was performed using Gene Set Analysis Toolkit software (www.bioinfo.vanderbilt.edu/webgestalt). The enriched GO categories are shown in red (hypergeometric; $P < 0.1$; red) and their nonenriched parents are shown in black.

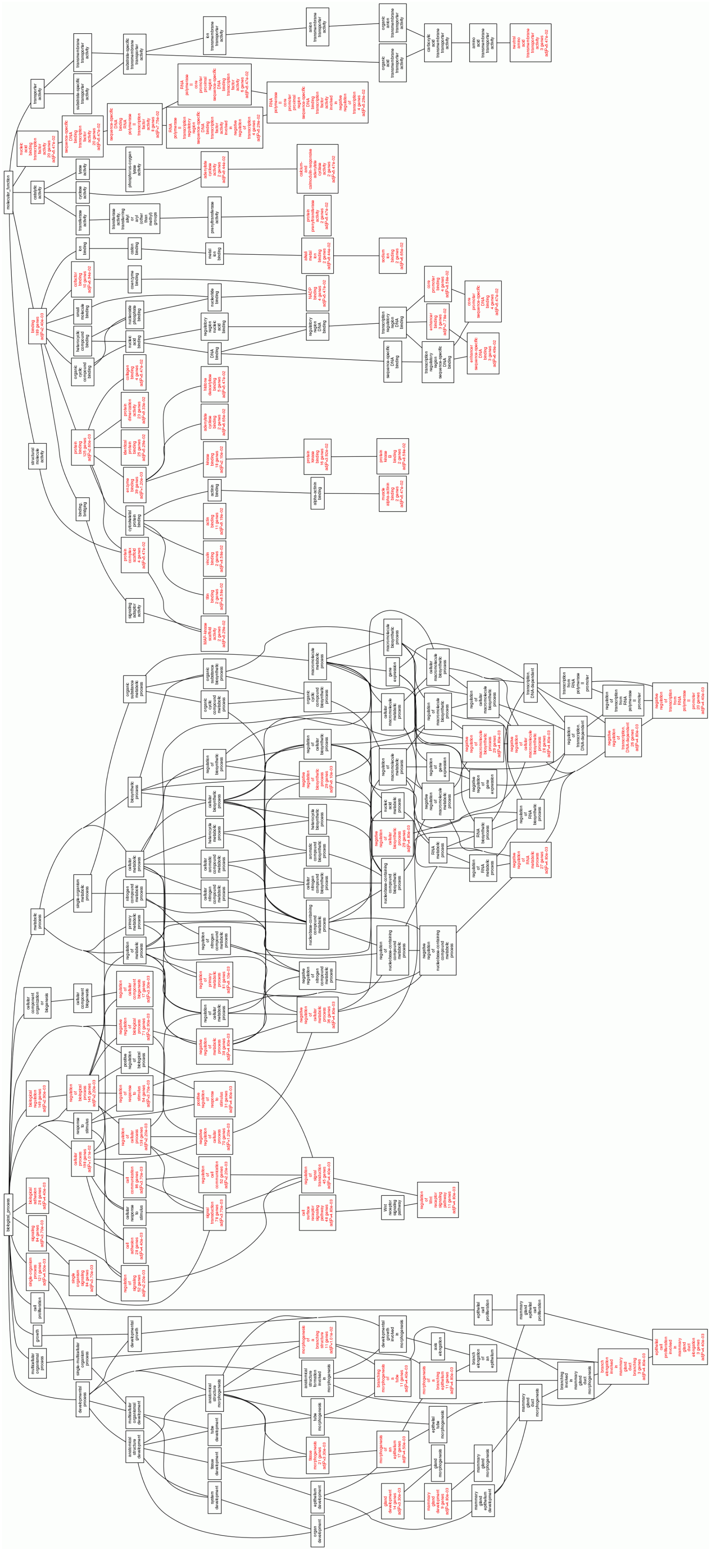

Supplementary figure S3 caption: Gene ontology (GO) analysis of connected genes in murine cells, which represent the tumor microenvironment (TME) of the melatonin-treated group. GO analysis was performed using Gene Set Analysis Toolkit software (www.bioinfo.vanderbilt.edu/webgestalt). The enriched GO categories are shown in red (hypergeometric; $P < 0.1$; red) and their nonenriched parents are shown in black.

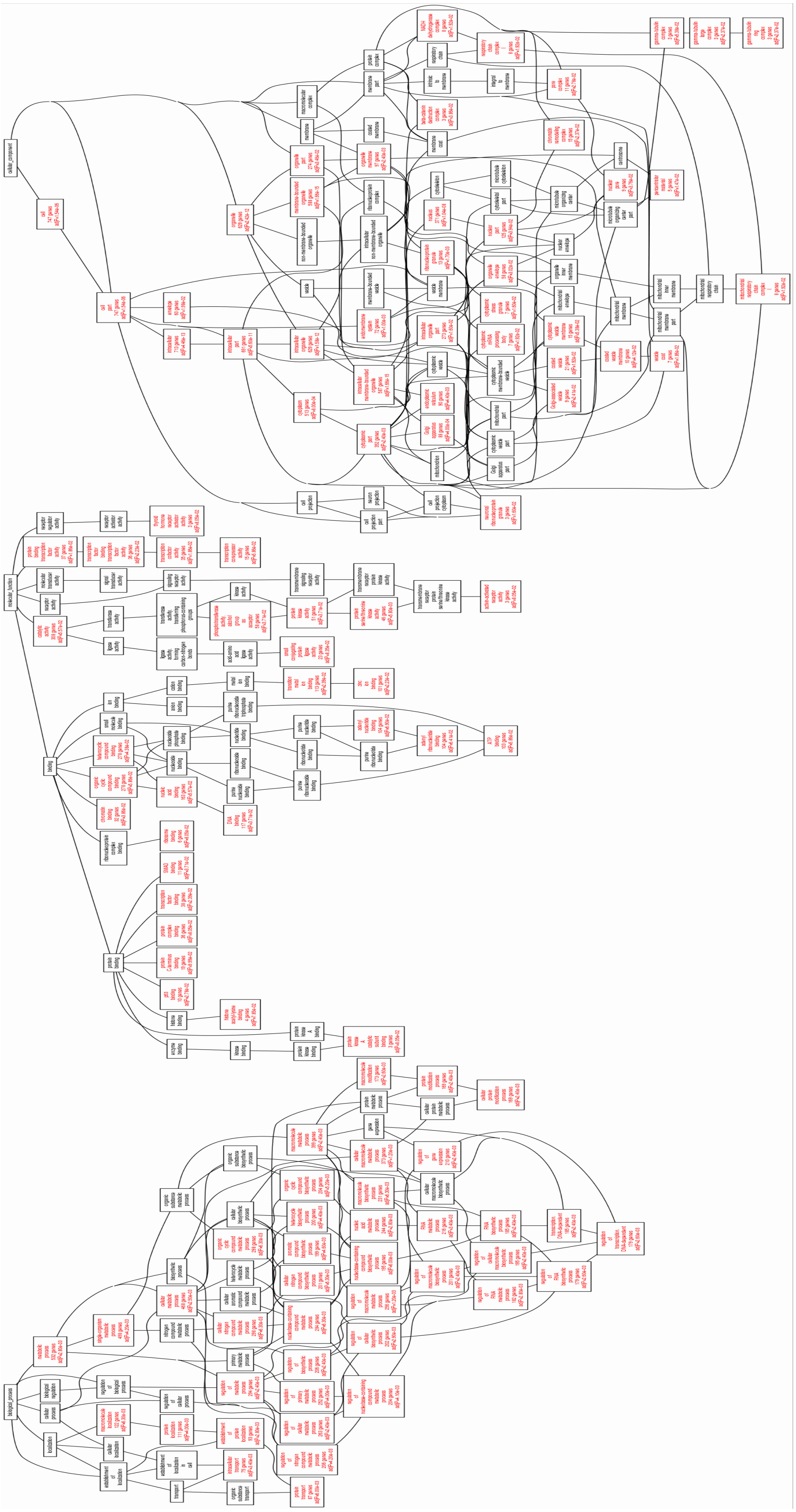

Supplementary figure S4 caption: Gene ontology (GO) analysis of connected genes in murine cells, which represent the tumor microenvironment (TME) of the control group. GO analysis was performed using Gene Set Analysis Toolkit software (www.bioinfo.vanderbilt.edu/webgestalt). The enriched GO categories are shown in red (hypergeometric; $P < 0.1$; red) and their nonenriched parents are shown in black.

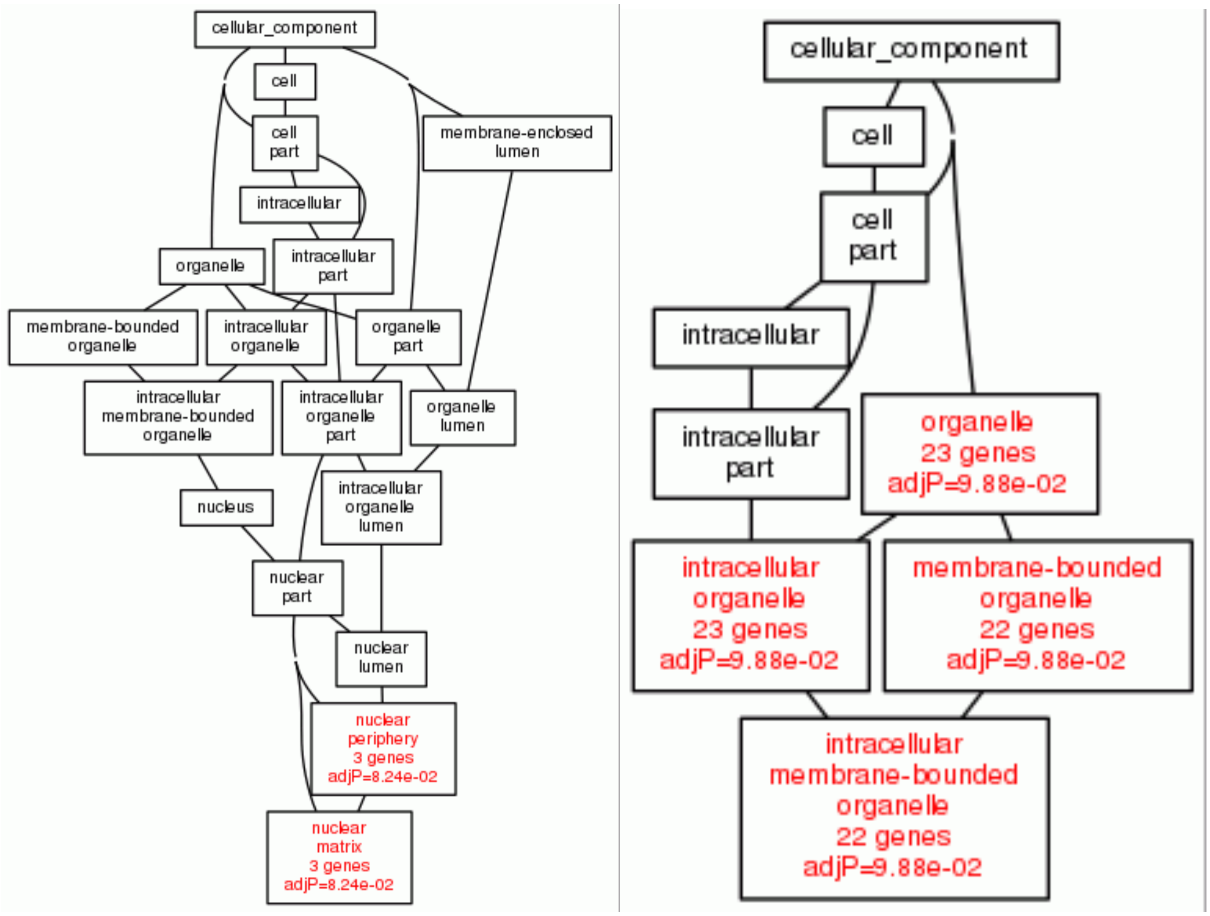

Supplementary Figure S5 caption: Gene ontology (GO) analysis of co-expressed genes modules altered by melatonin treatment in human tumors cells. **(Left)** Black module **(Right)** Magenta module. Pink module did not show enrichment. GO analysis was performed using Gene Set Analysis Toolkit software (www.bioinfo.vanderbilt.edu/webgestalt). The enriched GO categories are shown in red (hypergeometric; $P < 0.1$; red) and their nonenriched parents are shown in black.

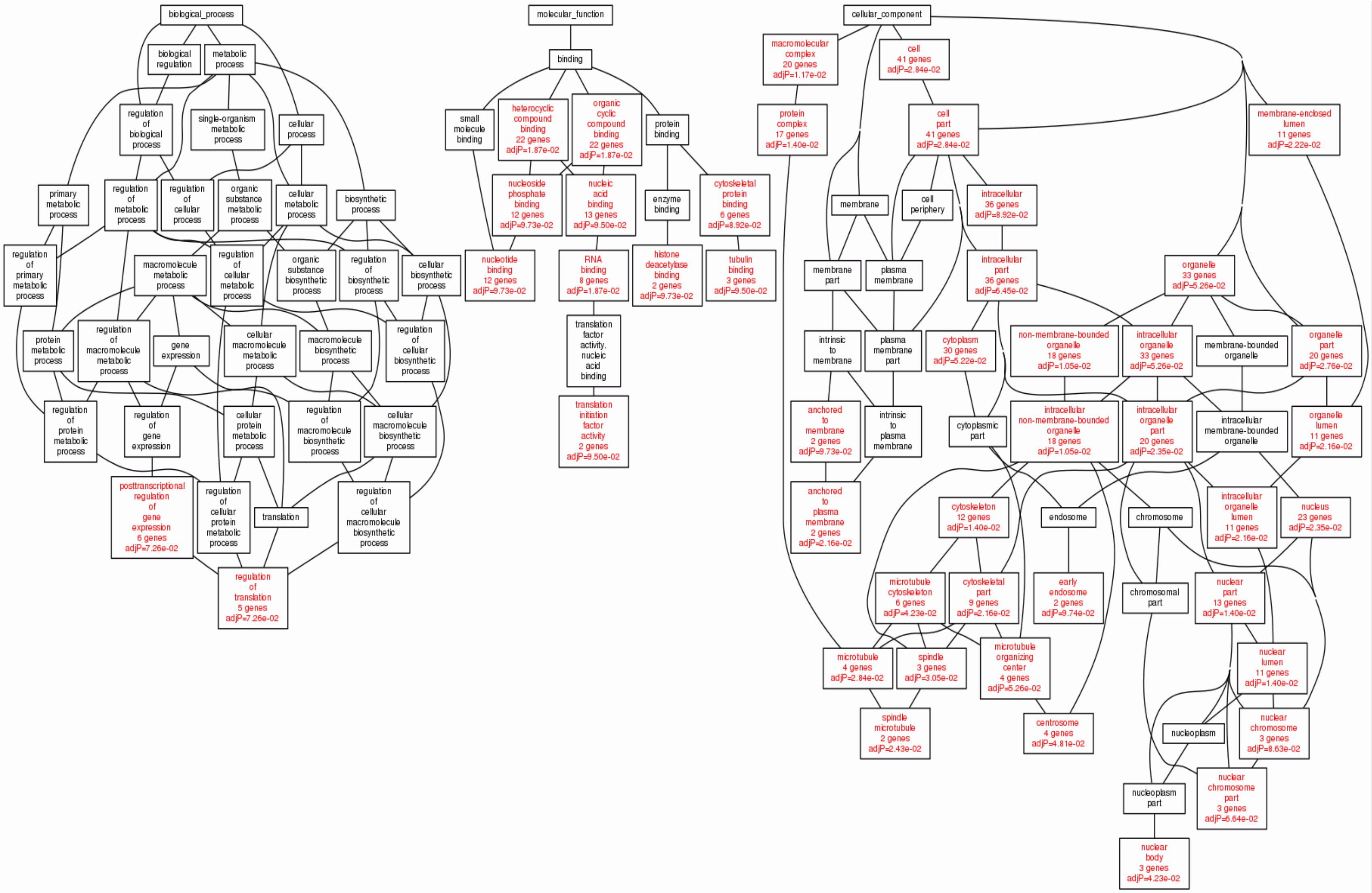
biological_process
biological regulation
metabolic process
regulation of biological process
single-organism metabolic process
cellular process
primary metabolic process
regulation of metabolic process
regulation of cellular process
organic substance metabolic process
cellular metabolic process
biosynthetic process
regulation of primary metabolic process
macromolecule metabolic process
regulation of cellular metabolic process
organic substance biosynthetic process
regulation of biosynthetic process
cellular biosynthetic process
protein metabolic process
regulation of macromolecule metabolic process
gene expression
cellular macromolecule metabolic process
macromolecule biosynthetic process
regulation of cellular biosynthetic process
regulation of protein metabolic process
regulation of gene expression
cellular protein metabolic process
regulation of macromolecule biosynthetic process
cellular macromolecule biosynthetic process
posttranscriptional regulation of gene expression 6 genes adjP=7.26e-02
regulation of cellular protein metabolic process
translation
regulation of cellular macromolecule biosynthetic process
regulation of translation 5 genes adjP=7.26e-02
molecular_function
binding
small molecule binding
heterocyclic compound binding 22 genes adjP=1.87e-02
organic cyclic compound binding 22 genes adjP=1.87e-02
protein binding
nucleoside phosphate binding 12 genes adjP=9.73e-02
nucleic acid binding 13 genes adjP=9.50e-02
enzyme binding
cytoskeletal protein binding 6 genes adjP=8.92e-02
nucleotide binding 12 genes adjP=9.73e-02
RNA binding 8 genes adjP=1.87e-02
histone deacetylase binding 2 genes adjP=9.73e-02
tubulin binding 3 genes adjP=9.50e-02
translation factor activity, nucleic acid binding
translation initiation factor activity 2 genes adjP=9.50e-02
cellular_component
macromolecular complex 20 genes adjP=1.17e-02
cell 41 genes adjP=2.84e-02
protein complex 17 genes adjP=1.40e-02
cell part 41 genes adjP=2.84e-02
membrane-enclosed lumen 11 genes adjP=2.22e-02
membrane
cell periphery
intracellular 36 genes adjP=8.92e-02
membrane part
plasma membrane
intracellular part 36 genes adjP=6.45e-02
organelle 33 genes adjP=5.26e-02
intrinsic to membrane
plasma membrane part
cytoplasm 30 genes adjP=5.22e-02
non-membrane-bounded organelle 18 genes adjP=1.05e-02
intracellular organelle 33 genes adjP=5.26e-02
membrane-bounded organelle
organelle part 20 genes adjP=2.76e-02
anchored to membrane 2 genes adjP=9.73e-02
intrinsic to plasma membrane
cytoplasmic part
intracellular non-membrane-bounded organelle 18 genes adjP=1.05e-02
intracellular organelle part 20 genes adjP=2.35e-02
intracellular membrane-bounded organelle
organelle lumen 11 genes adjP=2.16e-02
anchored to plasma membrane 2 genes adjP=2.16e-02
cytoskeleton 12 genes adjP=1.40e-02
endosome
chromosome
intracellular organelle lumen 11 genes adjP=2.16e-02
nucleus 23 genes adjP=2.35e-02
microtubule cytoskeleton 6 genes adjP=4.23e-02
cytoskeletal part 9 genes adjP=2.16e-02
early endosome 2 genes adjP=9.74e-02
chromosomal part
nuclear part 13 genes adjP=1.40e-02
microtubule 4 genes adjP=2.84e-02
spindle 3 genes adjP=3.05e-02
microtubule organizing center 4 genes adjP=5.26e-02
nuclear lumen 11 genes adjP=1.40e-02
spindle microtubule 2 genes adjP=2.43e-02
centrosome 4 genes adjP=4.81e-02
nucleoplasm
nuclear chromosome 3 genes adjP=8.63e-02
nucleoplasm part
nuclear chromosome part 3 genes adjP=6.64e-02
nuclear body 3 genes adjP=4.23e-02

Supplementary figure S6 caption: Gene ontology (GO) analysis of darkoliver co-expressed gene module altered by melatonin treatment in the murine cells, which represent the tumor microenvironment (TME). GO analysis was performed using Gene Set Analysis Toolkit software (www.bioinfo.vanderbilt.edu/webgestalt). The enriched GO categories are shown in red (hypergeometric; $P < 0.1$; red) and their nonenriched parents are shown in black.

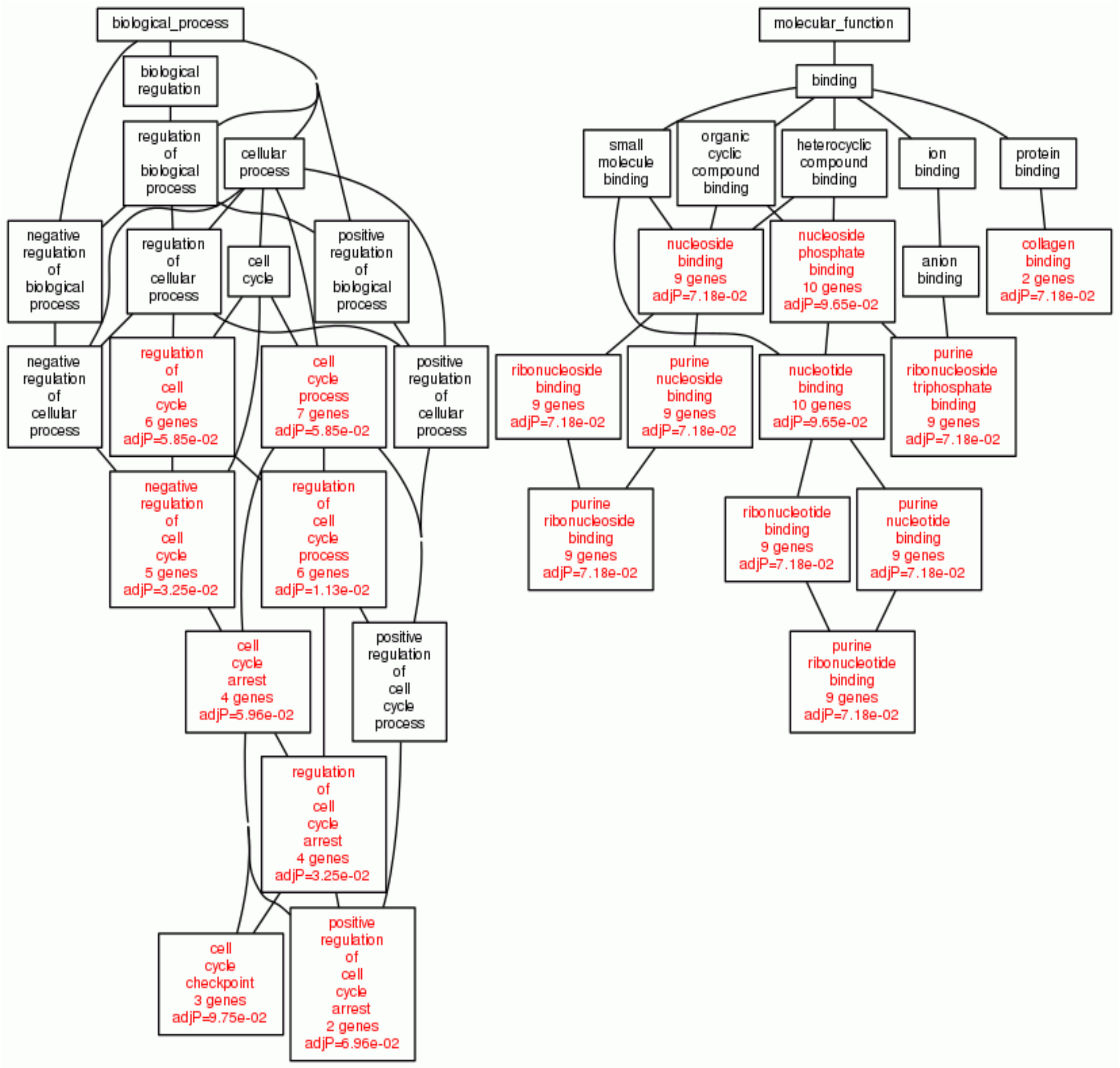

Supplementary figure S7 caption: Gene ontology (GO) analysis of yellowgreen co-expressed gene module altered by melatonin treatment in the murine cells, which represent the tumor microenvironment (TME). GO analysis was performed using Gene Set Analysis Toolkit software (www.bioinfo.vanderbilt.edu/webgestalt). The enriched GO categories are shown in red (hypergeometric; P < 0.1; red) and their nonenriched parents are shown in black.

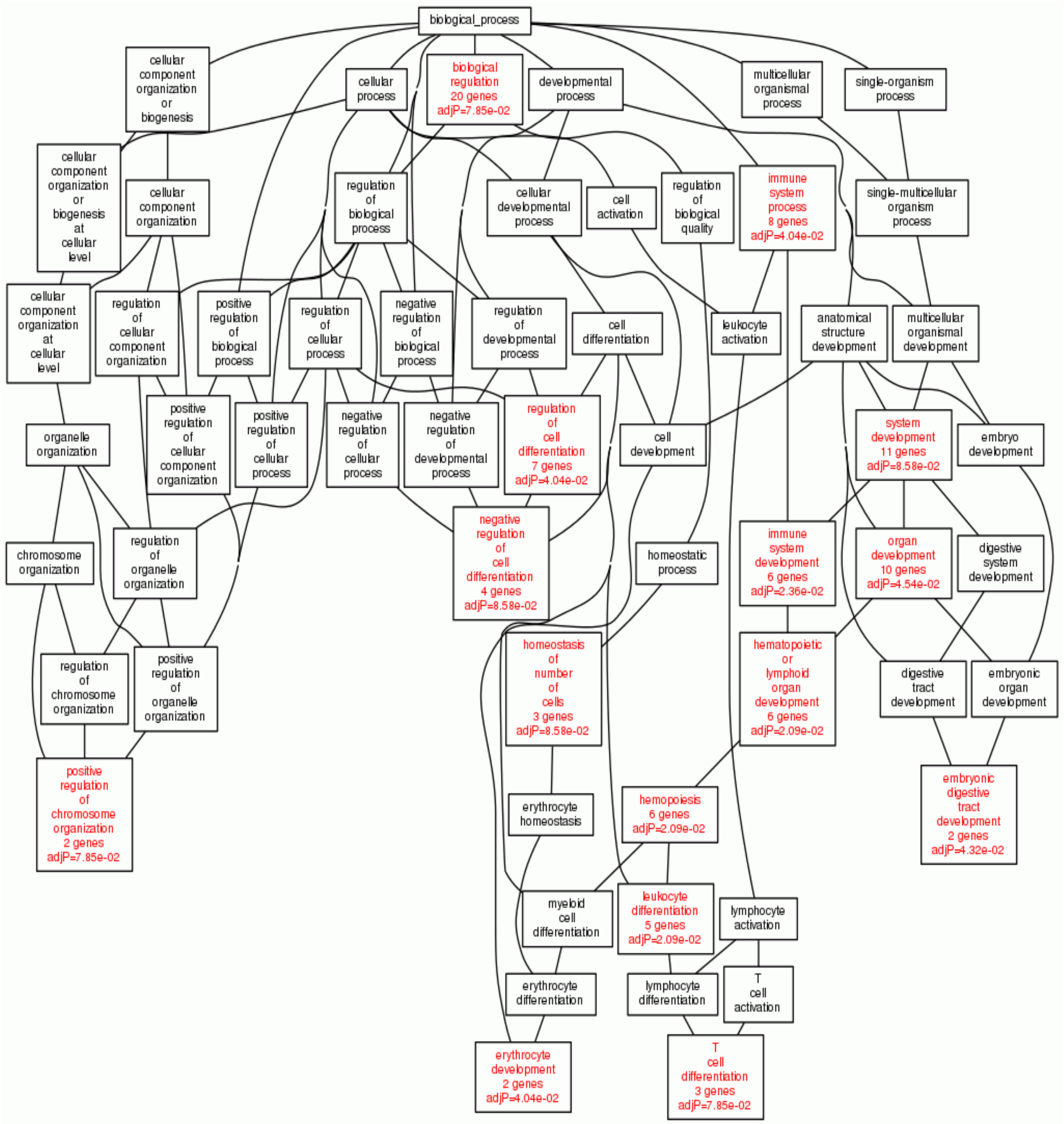

Supplementary figure S8 caption: Gene ontology (GO) analysis of orangered co-expressed gene module altered by melatonin treatment in the murine cells, which represent the tumor microenvironment (TME). GO analysis was performed using Gene Set Analysis Toolkit software (www.bioinfo.vanderbilt.edu/webgestalt). The enriched GO categories are shown in red (hypergeometric; P < 0.1; red) and their nonenriched parents are shown in black.

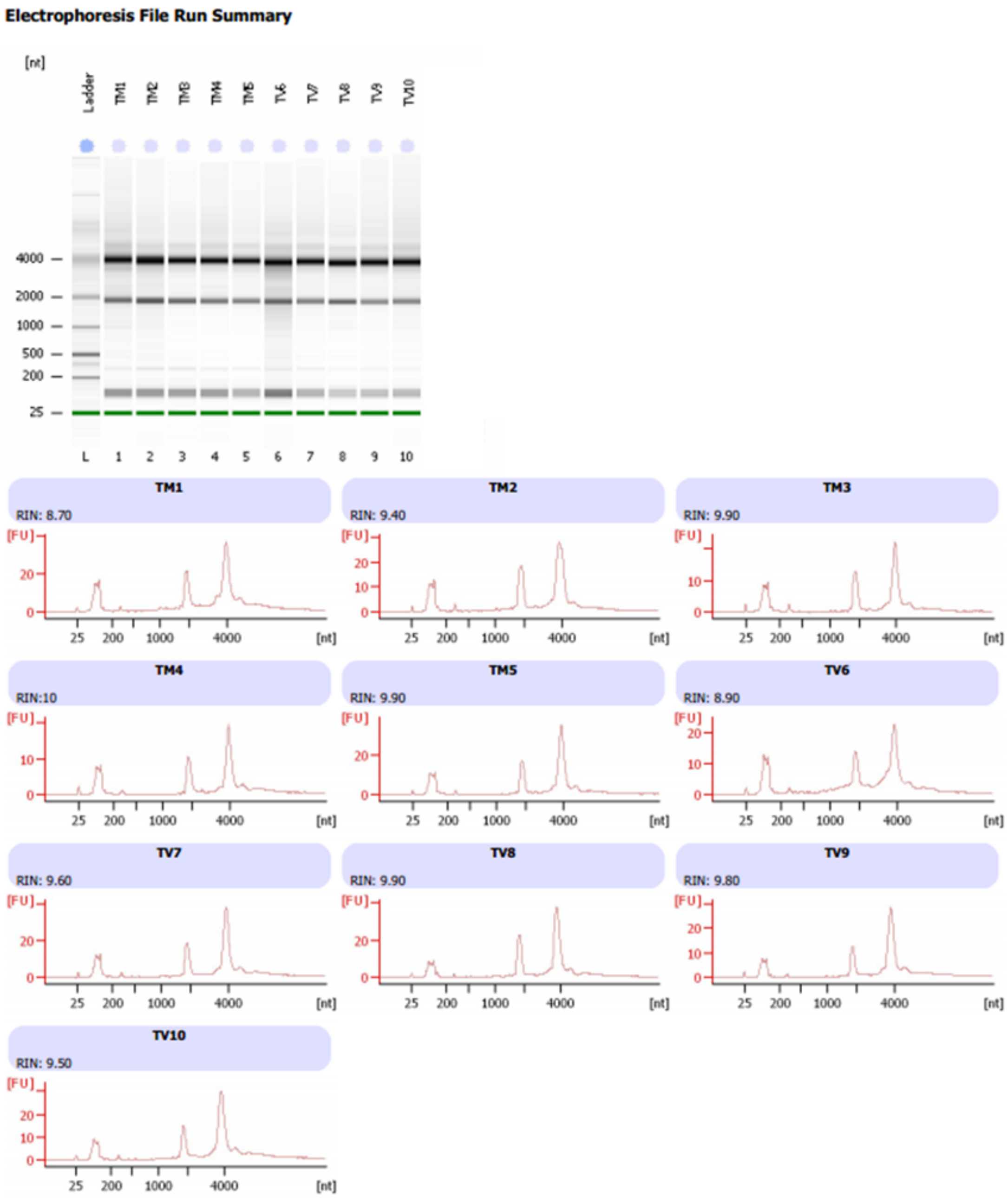

Supplementary figure S9. Bioanalyzer electropherograms showing the RNA quality for breast cancer samples. TM indicate the melatonin-treated samples and TV indicated vehicle-treated samples (control). All samples showed the RNA Integrity Number (RIN) higher than 8.7, confirming the RNA integrity and quality.
